# Sex differences in the traumatic stress response: the role of adult gonadal hormones

**DOI:** 10.1186/s13293-018-0192-8

**Published:** 2018-07-13

**Authors:** Apryl E. Pooley, Rebecca C. Benjamin, Susheela Sreedhar, Andrew L. Eagle, Alfred J. Robison, Michelle S. Mazei-Robison, S. Marc Breedlove, Cynthia L. Jordan

**Affiliations:** 1 Neuroscience Program, Michigan State University, 108 Giltner Hall, 293 Farm Lane, East Lansing, MI 48824 USA; 2 Department of Physiology, Michigan State University, 2201 BPS, 567 Wilson Rd, East Lansing, MI 48824 USA

**Keywords:** Single prolonged stress, HPA axis, PTSD, Post-traumatic stress disorder, Gonadectomy, Testosterone, Depression

## Abstract

**Background:**

Our previous study revealed that adult female rats respond differently to trauma than adult males, recapitulating sex differences in symptoms of post-traumatic stress disorder (PTSD) exhibited by women and men. Here, we asked two questions: does the female phenotype depend on (1) social housing condition and/or (2) circulating gonadal hormones?

**Methods:**

For the first study, the effects of single prolonged stress (SPS) were compared for females singly or pair-housed. For the second study, adult male and female rats were gonadectomized or sham-gonadectomized 2 weeks prior to exposure to SPS, with half the gonadectomized rats given testosterone. In addition to the typical measures of the trauma response in rats, acoustic startle response (ASR), and the dexamethasone suppression test (DST), we also used two other measures typically used to assess depressive-like responses, social interaction and sucrose preference. Glucocorticoid receptor (GR) expression in the hypothalamus was also examined.

**Results:**

We now report that the distinct trauma response of female rats is not influenced by social housing condition. Moreover, sex differences in the response to SPS based on ASR and DST, replicated in the current study, are independent of adult gonadal hormones. Regardless of hormonal status, traumatized males show a hyper-responsive phenotype whereas traumatized females do not. Moreover, testosterone treatment in adulthood did not masculinize the response to trauma in females. Notably, both sucrose preference and social interaction tests revealed an effect of trauma in females but not in males, with the effects of SPS on sucrose preference dependent on ovarian hormones. Effects of SPS on GR expression in the hypothalamus also depended on gonadal hormones in females.

**Conclusions:**

We propose that the trauma response for female rats is depressive in nature, recapitulating the female bias in PTSD for internalizing symptoms and major depression in contrast to the externalizing symptoms of males. Presumed core markers of PTSD (enhanced ASR and negative feedback control of corticosterone) are apparently relevant *only* to males and are independent of adult gonadal hormones. Such sex differences in trauma responding are likely determined earlier in life. We conclude that males and females show fundamentally different responses to trauma that do not simply reflect differences in resilience.

**Electronic supplementary material:**

The online version of this article (10.1186/s13293-018-0192-8) contains supplementary material, which is available to authorized users.

## Background

Post-traumatic stress disorder (PTSD) can develop after exposure to trauma and is associated with dysfunction in the normal stress response. There are also sex differences in trauma responding. Women are twice as likely as men to develop PTSD and tend to experience different symptoms and comorbidities than men [1–4]. The neurobiological basis for these differences is poorly understood due to a consistent and long-stranding trend to not study females. In the previous study (see companion paper), we replicated the well-established effects of traumatic stress in male rats, finding increases in both the acoustic startle response (ASR) and negative feedback control of the hypothalamic-pituitary-adrenal (HPA) axis [5], but we did not find the same responses in female rats. Rather, the same traumatic stressors had no apparent effect on either ASR or HPA negative feedback in female rats, aligning with clinical findings on women with PTSD [6–8]. We also found sex-specific changes in brain activation and glucocorticoid receptor (GR) expression after exposure to trauma that may explain the sex-specific effects on behavior and HPA negative feedback.

In the present study, we begin to explore the factors that determine these sex differences in the trauma response. We ask whether housing and/or testing conditions might play a role. In the first of two studies reported here, we compared the trauma response of females singly versus paired-housed, with ASR tested under bright ambient light, a factor shown to enhance acoustic startle for females [9]. In the previous study, rats were singly housed and tested under dim red light during their dark phase. Would these new conditions reveal an effect of single prolonged stress (SPS) on the ASR in females that was perhaps masked in the previous study by housing and/or light condition during the testing? Additionally, because traumatized females showed a reduced rather than an enhanced sensitivity to dexamethasone (DEX) in the previous study (see companion paper), suggestive of DEX non-suppression, a marker of depression in humans [10] and rodents [11], we added two new measures, social interaction and sucrose preference tests, that are typically used to assess depressive traits in rodents [12].

Because adult gonadal hormones dictate sex-specific corticosterone responses to acute stress in rodents [13–19], we also thought it plausible that adult gonadal hormones would play a decisive role in the sex-specific responses to traumatic stress. Thus, we compared the effects of stress in gonadally intact males and females to that of gonadectomized animals. We also asked whether testosterone (T) treatment of females could sex-reverse their response to trauma. In short, would T be sufficient to change the trauma response in females to that of males? Despite the well-established role of gonadal hormones in promoting sex differences in the brain and behavior [20], no studies have examined the possible role of gonadal hormones in sex differences in the traumatic stress response. To the extent that gonadal hormones have been considered as a predisposing factor in PTSD, the focus has been on the normal rise and fall of estrogen levels in women during the ovulatory cycle [21–23]. An entirely novel perspective to consider is whether higher levels of androgens confer protection against PTSD in males and mediate a male-specific response.

We now report that neither housing conditions nor bright light during testing of the ASR accounted for the sex difference in the trauma response. Regardless, SPS had no effect on ASR and negative feedback control of the HPA axis in females. We also replicated the sex difference in the trauma response reported in the previous paper (see companion paper), with males showing a hyper-responsive phenotype of increased ASR and enhanced negative feedback control of the HPA axis after trauma exposure that females did not show. Moreover, these sex differences in the trauma response were independent of sex differences in circulating levels of adult gonadal hormones. We also report for the first time female-specific effects of SPS on sucrose preference and social interaction, further suggesting a depressive-like phenotype for traumatized females, with sucrose preference depending on ovarian hormones. In sum, male rats show externalizing-type symptoms like men with PTSD (e.g., hyperarousal, aggression, and risk-taking behaviors) while female rats show internalizing-type symptoms like women with PTSD (e.g., sadness, loss of pleasure, and social difficulties) [3, 4, 24, 25]. Such differences are largely independent of adult gonadal hormones.

## Methods

### Animals

Eight-week-old adult Sprague-Dawley male and female rats (total *n* = 272) were purchased from Charles River (Wilmington, MA, USA) and pair-housed with 12 h reversed light-dark cycle, ad lib food and water. Cage bedding was changed weekly, and no testing was conducted on days of cage changes. All behavior tests were conducted in the dark phase ≥ 2 h of dark. Female rats were freely cycling and assigned to treatment groups without regard to estrous cycle stage. All animal procedures and care met or exceeded the NIH guidelines and were approved by Michigan State University Institutional Animal Care and Use Committee.

### Gonadectomy (GDX)

The day after arrival, rats were randomly assigned to a treatment group (sham + blank, GDX + blank, GDX + T). Cagemates were placed in the same surgery treatment group. Isoflurane anesthetized rats were gonadectomized or sham gonadectomized, using sterile technique. For sham GDX, gonads were accessed via either the scrotum (for males) or the abdominal cavity (for females). Under the same bout of anesthesia, Silastic capsules (1.6-mm internal diameter, 3.2-mm external diameter, 20-mm effective release length) filled with T or left blank were implanted s.c. at the back of the neck. Such implants provide a time-release of the hormone of interest for up to 4 weeks without the stress of daily injections [26]. Ketoprofen analgesia was given at the onset of the procedure and the following day. Rats recovered for 2 weeks and were handled daily during the second week.

### Study design

**Fig. 1:**
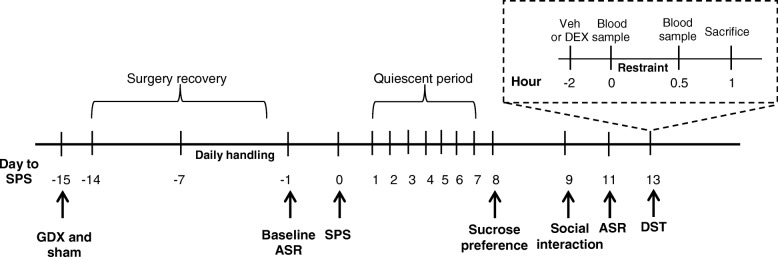
Timeline of experimental procedures. In the housing experiment, timeline begins with 7 days of daily handling (− 7) before single prolonged stress (SPS) and baseline acoustic startle response (ASR) testing the day before (− 1) SPS. In the gonadectomy experiment, GDX or sham surgery is performed 15 days before SPS, with daily handling 1 week before (− 7) SPS and baseline ASR testing the day before (− 1) SPS. Rats are left undisturbed for 1 week after SPS, and sucrose preference is assessed 8 days later, social interaction 9 days later, post-stress ASR 11 days later, and dexamethasone suppression test (DST) 13 days later

In the housing experiment, female rats were either single- or pair-housed and exposed to SPS or a no-stress control condition, in a 2 × 2 design with housing and stress as the two main factors. In the gonadectomy experiment, single-housed male and female rats were exposed to SPS or a no-stress control condition, in a 2 × 2 × 2 design with sex, stress, and hormone status as the three main factors. The experimental timeline (Fig. 1) began with 7 days of daily handling in the housing experiment, or GDX or sham-GDX 1 week before the 7 days of daily handling in the gonadectomy experiment. Baseline ASR testing was conducted the day before SPS, and rats were left undisturbed for 1 week after stress. Sucrose preference was assessed 8 days later, social interaction 9 days later, post-stress ASR 11 days later, and DEX suppression test (DST) 13 days later. Each experiment was conducted using multiple independent cohorts of rats, with equal numbers of rats representing all treatment groups (minimum of 2 rats per group) in a single cohort yoked through the entire experiment. The number of cohorts required was determined a priori by a power analysis using effect sizes obtained from preliminary studies to determine the number of rats required per group to achieve 0.80 statistical power, and data from each cohort in a single experiment were collapsed after confirming via ANOVA that showed no significant cohort effects.

### Acoustic startle response

Rats were placed in a Plexiglas tube attached to an accelerometer inside a soundproof chamber (SR-Lab, San Diego Instruments, CA, USA) and allowed to acclimate for 5 min (68 dB background noise) before delivery of a startle stimulus (50 ms burst of 110 dB white noise every 30 s for 15 min), as previously described [5]. The chamber and Plexiglas tube were cleaned with 70% ethanol between each test. In the housing experiment, the startle chamber was brightly lit with an ambient light in the ceiling of the chamber. In the gonadectomy experiment, the chamber was dark. Peak whole-body startle response was recorded every 1 ms for 100 msec, beginning with each startle stimulus. The average peak value for each rat was normalized to body weight. Baseline measures were taken on the day before stress exposure, with rats randomly selected and counterbalanced by group. Cagemates were tested simultaneously in two separate chambers. Rats were then assigned to control or stress groups so that each group had equal average ASR [27]. ASR testing was repeated 8 days after stress exposure with individual rats tested in the same order as baseline ASR.

### SPS paradigm

In the housing study, half the rats were singly housed immediately before exposure to SPS or control and the other half remained pair-housed with their original cagemate. In the gonadectomy study, all rats were singly housed immediately before exposure to SPS or control conditions. SPS consists of a psychological stressor (2 h tube-restraint stress, Braintree Scientific, Braintree, MA, USA), a physiological stressor (in 20 min, group was forced to swim in 24 °C water; *n* = 6 same sex rats per 75 l tub with 28 cm water depth), and a chemical stressor (brief exposure to diethyl ether until immobile and lacking toe-pinch response), as previously described [28]. Rats were given a 15-min rest period in their home cages after they were forced to swim before ether exposure. Ether is used as a chemical/endocrinological stressor to recapitulate an important phenotypic aspect of PTSD, not as an anesthetic. Control rats were also similarly removed from the vivarium for 2.75 h. Rats were left undisturbed for 1 week after SPS, a requisite delay for the long-lasting PTSD responses to develop [5].

### Sucrose preference test

To measure anhedonia, rats were given access to two bottles containing either 0.8% sucrose solution or tap water for 24 h. To account for any circadian influences and side-preferences, the two water bottles were presented halfway through the rats’ dark cycle and the bottle position switched after 12 h, as previously described [29]. The bottles were initially weighed and a final weight taken at 24 h. Sucrose preference was calculated as percent of total intake. To minimize the potential influence of metabolic factors and acute stress, food and water were available ad lib.

### Social interaction test

Each test rat was placed in a clear Plexiglas box (60 cm^3^) at the end opposite an interaction zone measuring 8 cm out from the edge of an empty circular wire enclosure. After 2.5 min, a probe rat matched for sex and age was placed inside the wire enclosure for an additional 2.5 min, as previously described [30]. Behavior was videotaped, and the videos were analyzed blind for measures of social interaction. Social interaction behaviors analyzed included latency to first enter the interaction zone and time spent in the interaction zone. To assess social interaction, we used the measure “latency to enter the interaction zone” rather than the more typical measure of total social interaction time because of the established sex-specific effects of stress on latency [31]. Data from this measure (latency to enter the interaction zone containing a social target) was normalized to the latency to enter this same interaction zone without a social target (the empty wire enclosure), which controlled for individual differences in generalized motor activity, another sexually differentiated attribute [32–35]. Thus, we feel that social interaction latency is the valid and appropriate measure to assess how prior exposure to SPS might influence the inclination of males and females to socially interact.

### Dexamethasone suppression test

To assess the strength of negative feedback control of the HPA axis in response to acute restraint stress, the DST was performed 2 weeks post-SPS, as previously described [5]. Rats from each group were randomly assigned to receive either DEX or vehicle. Dexamethasone (Sigma-Aldrich, St. Louis, MO, USA) was dissolved with ethanol and diluted to 5% in sterile saline. Low-dose DEX (0.05 mg/kg, i.p.) or vehicle was administered 2 h prior to 30 min tube-restraint. Tail-nick blood samples were collected at 0 and 30 min of restraint. Blood samples were collected in the rats’ dark phase, matching the time of day across experimental groups. Plasma corticosterone (CORT) levels were determined using an enzyme immunoassay kit. Rats were overdosed with pentobarbital (i.p.) after 30 min of restraint, then intracardially perfused with saline and 4% buffered paraformaldehyde, with brains harvested for staining.

### Glucocorticoid receptor immunohistochemistry (IHC)

The brains were sectioned and labeled for GR using a peroxidase ABC kit (Vectastain Elite ABC Kit, Vector Labs, cat. no. PK-6200), and only rats that received vehicle injections (and not DEX) were used to evaluate GR expression in the brain. A GR primary antiserum (1:2500; rabbit polyclonal IgG; Santa Cruz Biotech, cat. no. M-20, Dallas, TX, USA) and diaminobenzidine was used to visualize GR expression. Specificity of GR staining was confirmed by observing the expected regional staining (e.g., in the dentate gyrus and CA1 but not CA3) and observing a loss of nuclear staining when the GR antiserum was preadsorbed with the immunizing peptide. Microscope analysis was conducted on a Zeiss Axioplan light microscope equipped with a video camera and MBF Stereo Investigator software (MBFBioscience, Williston, VT, USA). The number of cFos+ or GR+ neurons within specific brain regions was counted blind from four comparable sections per rat using unbiased stereological methods, and the number of immunopositive cells per cubic millimeter was quantified for each region by dividing the total number of cells counted within a region by the measured volume of that region. Anatomical position was determined using a stereotaxic atlas.

### Statistical analysis

All data were collected by experimenters blind to treatment group. All rats from all cohorts in a single experiment were included in the final statistical analysis, excepting the following exclusion criteria determined a priori: (1) if baseline CORT was not reduced in a DEX-treated rat *and* experimenter notes confirm a partial/problematic injection, that individual was excluded from *only* the DST analysis, (2) if CORT levels did not increase (either 0 or negative change) after 30 min of restraint, that individual was excluded *only* from the DST analysis, and (3) if water intake was significantly higher than group average *and* experimenter notes confirm a potential bottle leakage, that individual was excluded from *only* the sucrose preference analysis. These exclusion criteria resulted in the exclusion of 0–2 individuals from each treatment group for the DST. After confirming equal variance between treatment groups, two-, three- or four-way ANOVAs were run for comparisons in rats. Analysis was first conducted on only sham + blank rats to assess whether previously identified sex differences were replicated. Then, the effects of GDX and hormone treatment were assessed within each sex. For brain measures, if no main effect or interactions of hemisphere was present, data were collapsed across hemisphere. See Additional files 2, 3, 4, and 5 for all statistical tests performed. The conservative Bonferroni test was used to correct for multiple tests to hold the probability of a type I error at 0.05.

## Results

### Neither housing nor bright lights affect the female response to SPS

We first compared the effects of SPS in females that were single- or pair-housed, wanting to know whether housing was a factor contributing to the female-specific response to SPS. Because our results based on single-housed males replicated those of group-housed males [36], we did not include a cohort of males in this study. Tests were run under bright ambient light because it can enhance acoustic startle for females [9]. All other conditions were the same as in our previous study (see companion paper). Even under bright lights, SPS did not affect ASR in females, nor did the null effect of SPS on ASR depend on housing condition (Additional file 1a). Likewise, DEX failed to block a stress-induced CORT response in SPS-exposed females regardless of housing condition (Additional file 1b), unlike the male-typical response to SPS. DEX again lowered baseline CORT levels to near zero in all groups, confirming the effectiveness of the exogenous hormone, and neither housing condition nor SPS affected baseline CORT levels. Thus, female rats show a unique response to SPS that is distinct from males, independent of housing condition (group versus single) and ASR light testing condition; neither acoustic startle nor HPA negative feedback revealed an effect of SPS in female rats.

Social interaction was affected by SPS in females, although the direction of effect depended on housing. For pair-housed females, SPS increased the latency to approach a novel rat (social target), suggestive of an anxious phenotype, but for singly housed females, SPS decreased the latency to approach a novel rat, perhaps reflecting an inclination to seek social support (Additional file 1c). Because neither housing nor SPS affected the latency to approach the empty enclosure (lacking a social target) (Additional file 6a), the effect of SPS was on social interaction per se. Total social interaction time was not sensitive to either the effects of SPS or housing condition (Additional file 6b). Our data indicate that social interaction latency is a sensitive readout of the traumatic stress response for female rats exposed to SPS. In the next study, we ask whether SPS affects social interaction in males as well as females. Contrary to expectation, SPS had no effect on sucrose preference in either housing condition (Additional file 1d), questioning the extent to which the female response to SPS is a depressive, anhedonic-like response. Body weight was not affected (Additional file 1e). For experimental consistency, we continued to use single housing in subsequent studies.

### Gonadally intact male and female rats respond differently to SPS

**Fig. 2:**
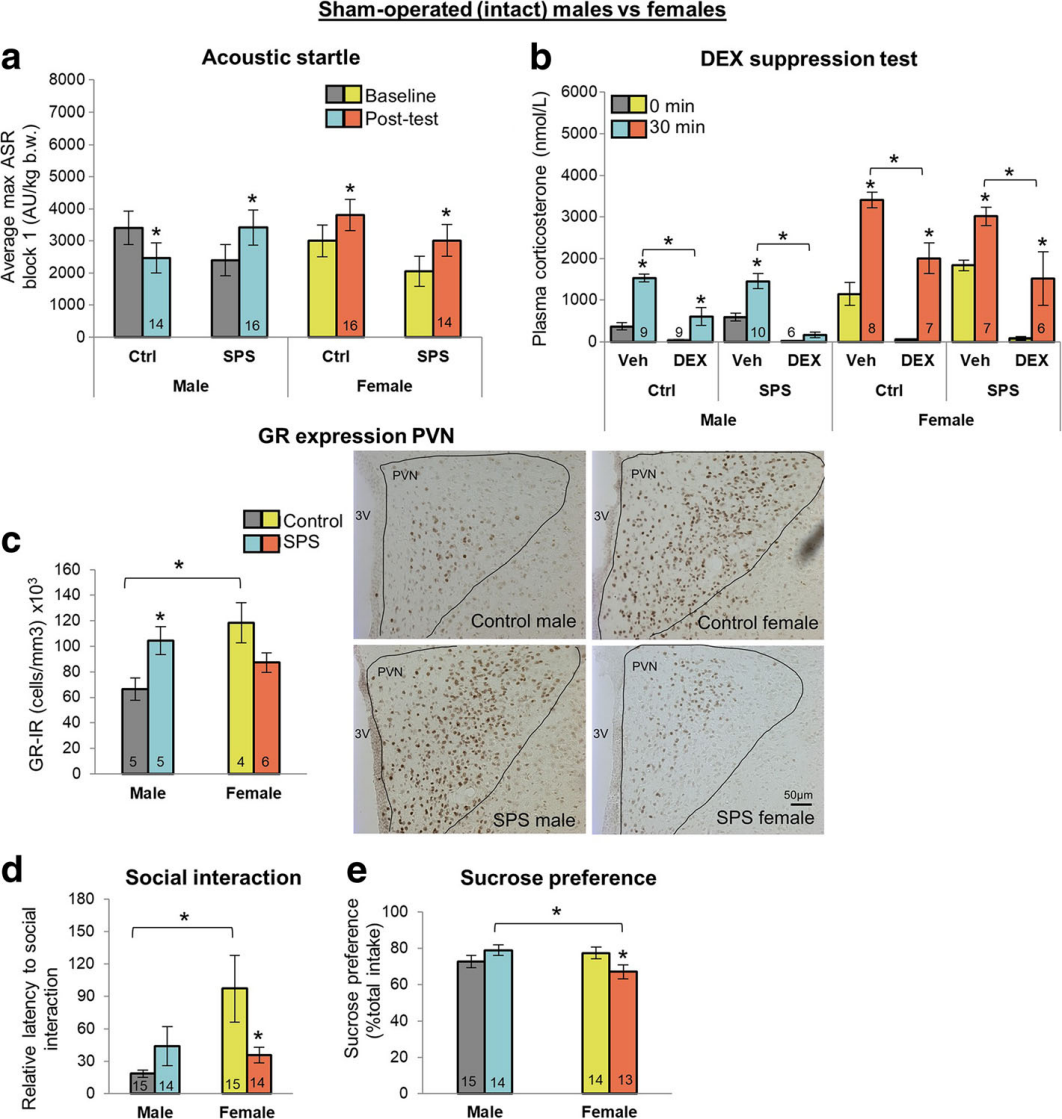
SPS affects males and females differently in sham gonadectomized rats. **a** SPS enhanced acoustic startle response (ASR) *only* in males. While females showed significant increases in ASR in the post-test compared to baseline, this effect was not specific to SPS exposure as it is for males. Control males showed a habituation to the acoustic stimulus in the post-test compared to baseline. **b** SPS enhanced negative feedback control of the HPA axis *only* in males. Note that while DEX completely blocked the increase in CORT levels induced by acute restraint stress in SPS-exposed males, it did not block this increase in SPS-exposed females, nor did DEX block this increase in control males and females. SPS did increase *baseline* CORT levels in females but had no effect on baseline CORT in males. DEX drove baseline CORT levels to near zero in all four groups, confirming its efficacy in both sexes. **c** SPS increased glucocorticoid receptor (GR) expression in the paraventricular nucleus of the hypothalamus (PVN) in males, while the decrease in GR expression in SPS females fell short of significance (*P = .056*). Control females had more GR+ PVN neurons than control males. Representative photomicrograph is shown with the right PVN outlined and GR+ neurons visible with dark staining. **d** SPS affected social interaction and **e** sucrose preference *only* in females, significantly decreasing their latency to approach a novel conspecific and preference for sucrose. Control females also took longer than control males to approach a novel rat. Data was presented as mean ±SEM. Significance set at *P* < .05 (*) for planned pairwise comparisons (Bonferroni). See Additional file 3 for full statistics

We first compared sham-operated males and females and found comparable sex-specific responses to SPS as previously reported (see companion paper), indicating that sham surgery itself did not alter the pattern of differences (Fig. 2a–c). SPS-exposed males again showed the expected increases in the ASR and HPA negative feedback, but SPS-exposed females did not, while GR expression in the periventricular nucleus of the hypothalamus (PVN) was increased by SPS in males and decreased by SPS in females. However, note that both SPS and control females showed a significant ASR increase in the post-test compared to baseline, in contrast to the previous study in which neither group of females showed a change in ASR (see companion paper). The female increase in ASR could be due to the stress of the sham-surgery procedure, which is a major abdominal surgery for females but only a minor external incision for males. Nonetheless, SPS still did not differentially affect ASR in these sham-operated females as it did for males. We also replicated an effect of SPS on social interaction latency in females (Fig. 2d, Additional file 6c, d) and found no effect of SPS on this measure in males. Unlike in the housing experiment, SPS significantly decreased sucrose preference in females (Fig. 2e). Sucrose preference in males was unaffected by SPS. These data suggest that both sucrose preference and social interaction capture effects of SPS on behavior in females. Interestingly, males appear *resilient* to trauma based on these particular measures. These sex-specific effects of SPS depended neither on body nor adrenal gland weight (Table 1).

**Table 1:** SPS did not affect body or adrenal weight in sham-operated (intact) males and females

Group (*n* = 16/group)		Body weight in grams (SEM)		Adrenal weight in grams (SEM)
		Baseline	Post-test	
Male	Ctrl	304.1 (6.5)	332.8* (8.4)	0.19 (0.02)
	SPS	309.7 (6.4)	338.3* (6.2)	0.17 (0.01)
Female	Ctrl	199.4 (2.7)	213.0* (2.9)	0.34 (0.02)
	SPS	199.4 (3.8)	209.4* (4.1)	0.37 (0.03)

Rat body weights were collected before undergoing SPS or control conditions (baseline) and before the dexamethasone suppression test (post-test). Adrenal weights were collected after sacrifice. SPS did not affect body weight, and all rats gained weight over the course of the experiment (*, vs baseline). As expected, female body weight was lower, but adrenal weight higher, than that of males. Data was presented as mean ±SEM. Significance set at *P* < .05 (indicated by asterisk) for planned pairwise comparisons (Bonferroni). Refer to Additional file 3 for full statistical results

### Effects of SPS in males are largely independent of testicular hormones

**Fig. 3:**
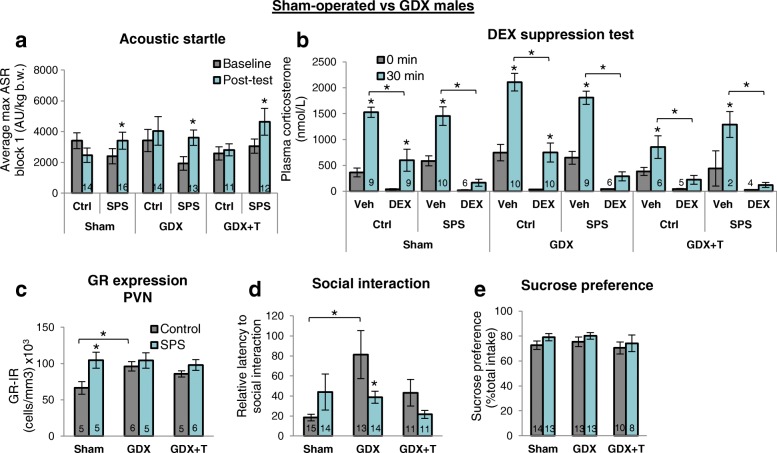
Effects of SPS in males are largely independent of adult testicular hormones. **a**, **b** Changes in gonadal hormone status did not affect the ability of SPS to enhance the ASR or negative feedback control of stress-induced CORT levels in male rats. In each hormonal condition, DEX inhibited the rise in CORT levels 30 min after restraint stress only in SPS males, with the exception of control males that were gonadectomized and treated with testosterone (GDX + T), possibly reflecting the small sample size of this particular group. **c** While SPS increased GR expression in the male PVN, this effect was not apparent in GDX males. Because GDX alone also increased the number of GR+ PVN neurons, this effect may have masked a genuine effect of SPS. Because T treatment did not reverse this effect, other testicular hormones may normally inhibit GR expression in the PVN. **d** GDX increased the latency of control, but not SPS, males to approach a novel rat, whereas SPS per se appeared to have no effect on this measure. These data highlight the possibility that gonadal hormones may mask real effects of trauma or spuriously introduce apparent effects of trauma, simply because baseline measures are influenced by hormones independent of the trauma itself. **e** Neither SPS nor hormone status affected sucrose preference in males. Data was presented as mean ±SEM. Significance set at *P* < .05 (*) for planned pairwise comparisons (Bonferroni). See Additional file 4 for full statistics

Overall, we find that male rats castrated as adults respond to SPS much like gonadally intact males. To the extent that adult testicular hormones influenced our measures, they were independent of SPS. Specifically, gonadal status did not alter the effect of SPS on the ASR in males (Fig. 3a). Likewise, the DST revealed the same pattern of differences in castrated (GDX) males as in gonadally intact (sham) males (Fig. 3b). Both groups showed an exaggerated DEX suppression after exposure to SPS. Note that in each case, CORT levels are comparable before and after restraint stress in the presence of DEX, whereas in controls not exposed to SPS but given DEX, CORT levels are appreciably higher after acute restraint stress than before. Removing the testes did however significantly increase both baseline and restraint-induced CORT levels in control males, which was reversed by T treatment (GDX + T), as previously reported [17]. However, T treatment also seemed to mask an effect of SPS on HPA negative feedback since control males had comparably low, near baseline, CORT levels after restraint stress in the presence of DEX as SPS males. This outcome may reflect the fact that exogenous T does not faithfully recapitulate normal levels of endogenous T. Regardless, these data indicate that the effect of SPS in males on the ASR and negative feedback control of stress-induced CORT levels does not depend on endogenous testicular hormones in adulthood.

SPS and castration each significantly increased the number of GR+ neurons in the PVN (Fig. 3c). Specifically, SPS increased GR expression in gonadally intact but not castrated males while castration increased GR expression in control but not SPS-exposed males. This complex interaction between SPS and gonadectomy meant that the effect of SPS in gonadally intact males was no longer apparent in castrated males. These results indicate that testicular hormones normally regulate GR expression in the PVN, and depending on gonadal status, an effect of SPS on GR expression in the PVN may or may not be detected. However, T treatment did not reverse this effect of castration. Since other measures (e.g., Fig. 3d, described below) confirm that the T capsules were effective, these data raise the question of whether other testicular factors and/or hormonal responses to castration may regulate GR expression in the PVN of males.

While testicular hormones had no effect on sucrose preference in adult males (Fig. 3e), they did affect social interaction (Fig. 3d, Additional file 6e, f), revealing a significant effect of SPS in GDX males that is not apparent in gonadally intact males. Castration of control males increased the latency to approach a novel male (Fig. 3d) while decreasing the latency to approach the empty interaction chamber (Additional file 6e), pushing both measures toward a more feminine phenotype. That an effect of SPS is detected on social interaction latency in castrated but not gonadally intact males suggests that androgens may normally mask an effect of SPS on this measure in males. Total social interaction time, on the other hand, was not sensitive to either the effects of SPS or hormone group (Additional file 6f). Body and adrenal weight in males were unaffected by any treatment (Table 2). Behind a main effect of GDX on body weight was a significant lower body weight in SPS-castrated males compared to SPS sham males, but this effect was present at baseline before any testing, indicating that particular group had smaller males independently of surgery or SPS.

**Table 2:** Neither SPS nor hormone status affected body weight or adrenal weight in males

Group		Body weight in grams (SEM)		Adrenal weight in grams (SEM)
		Baseline	Post-test	
Sham	Ctrl	304.1 (6.5)	332.8* (8.4)	0.19 (0.02)
*n* = 16/gp	SPS	309.7 (6.4)	338.3* (6.2)	0.17 (0.01)
GDX	Ctrl	292.2 (7.2)	319.6* (6.6)	0.21 (0.02)
*n* = 16/gp	SPS	282.9 (5.5)	306.4* (6.1)	0.21 (0.01)
GDX + T	Ctrl	289.3 (7.9)	316.3* (9.2)	0.20 (0.01)
*n* = 12/gp	SPS	291.7 (9.0)	313.8* (7.4)	0.19 (0.004)

Rat body weights were collected before undergoing SPS or control conditions (baseline) and before the dexamethasone suppression test (post-test). Adrenal weights were collected after sacrifice. Neither SPS nor gonadecomty (GDX) or testosterone (T) affected body or adrenal weight in males and all rats gained weight over the course of the experiment (*, vs baseline). Data was presented as mean±SEM. Significance set at *P* < .05 (indicated by asterisk) for planned pairwise comparisons (Bonferroni). Refer to Additional file 4 for full statistical results

### Effects of SPS in females are largely independent of ovarian hormones

**Fig. 4:**
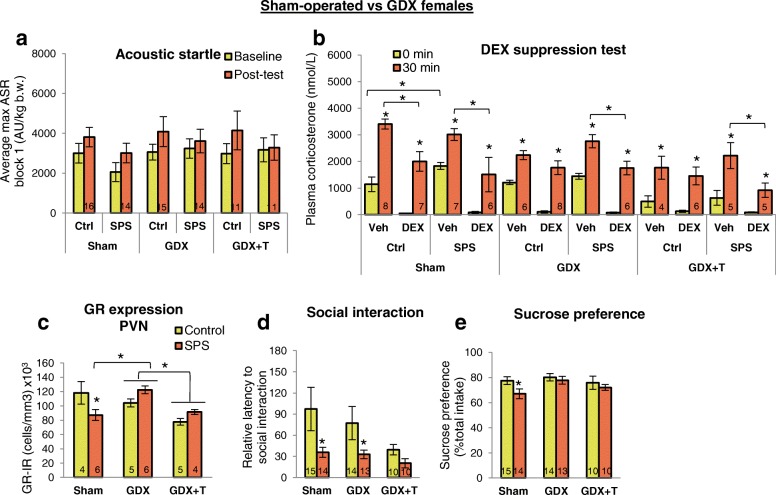
Effects of SPS in females are largely independent of ovarian hormones. **a**, **b** SPS had no effect on ASR or negative feedback control of CORT in females, regardless of gonadal hormone status. CORT levels were sensitive to ovarian hormones in vehicle-treated *control* females, with GDX significantly decreasing the CORT response to restraint stress. T did not reverse this effect. **c** SPS decreased GR expression in the PVN of sham females, an effect abolished by GDX, suggesting that ovarian hormones may increase the sensitivity of the PVN to stress. T treatment reduced GR expression overall in the PVN but did not restore the effect of SPS on this measure, suggesting that ovarian hormones regulate GR expression in the PVN via estrogen and/or progesterone receptors. **d** SPS decreased the latency to approach a novel rat in both sham and GDX females, indicating the effect of SPS on social interaction is independent of adult ovarian hormones. However, T treatment reduced the latency of control but not SPS females to approach a social target, such that the effect of SPS on this measure was no longer apparent. **e** SPS decreased sucrose preference in sham females, an effect abolished by GDX, implicating ovarian hormones in this female-specific response to SPS. Data was presented as mean ±SEM. Significance set at *P* < .05 for planned pairwise comparisons (Bonferroni). *, vs baseline same group; $, vs sham same group. See Additional file 5 for full statistics

Regardless of ovarian hormone status, ASR is not influenced by SPS in females (Fig. 4a). We also asked whether T treatment could convert the female response to that of males but find no effect of adult T treatment on the ASR in females (Fig. 4a). Moreover, neither gonadal status nor T treatment influenced the results of the DST in SPS-exposed females (Fig. 4b). As expected, 30 min of acute restraint stress significantly increased the level of CORT in all vehicle-treated groups, although the level was significantly reduced in *both* groups of gonadectomized control females (blank and T-treated) compared to sham control females. That T did not reverse the effect of ovariectomy suggests that deficits in estrogens and/or progestins underlie in the blunted CORT response to restraint stress in GDX females. Importantly, the same low dose of DEX that blocked a significant increase in CORT in SPS-exposed males after 30 min of restraint stress did *not* block this increase in CORT in SPS-exposed females, regardless of gonadal status or T treatment, while still lowering both baseline (0 min) and post-restraint stress CORT levels overall, demonstrating that DEX was working. As expected, T treatment significantly decreased baseline CORT levels in females [14]. These data indicate that the enhanced CORT suppression of traumatized males is not a characteristic feature of the trauma response of females, regardless of adult gonadal hormone status, raising questions about “how,” “what,” and “when” such sex differences are programmed.

Interestingly, the effect of SPS on the number of GR+ neurons in the PVN did depend on adult ovarian status. Without ovarian hormones, SPS had no effect on GR expression in the PVN as opposed to decreasing their number in gonadally intact females (Fig. 4c). Because ovariectomy per se did not affect the number of GR+ neurons in the PVN, ovarian hormones likely regulate how sensitive PVN neurons are to stress, with stress influencing GR expression in this brain region when ovarian hormones are present. The effect of SPS on social interaction did not depend on ovarian hormones since SPS shortened the latency to approach a novel female, replicating previous results (Additional file 1c), regardless of gonadal status (Fig. 4d). T treatment also reduced social interaction latency in control (non-SPS) females, causing the effect of SPS on this measure in GDX females to drop out. Latency to enter the interaction zone containing only an empty chamber was unaffected by SPS in females except in the presence of T, when approach time was increased by SPS compared to SPS-exposed females in the other two hormone groups (Additional file 6g). Total social interaction time was not sensitive to either the effects of SPS or hormone group (Additional file 6h). SPS significantly and selectively reduced sucrose preference in sham-operated females (Fig. 4e), an effect that was eliminated by ovariectomy. Because T did not reverse the effect of adult ovariectomy, estrogens and/or other ovarian factors likely mediate the effect of SPS on sucrose preference. Again, SPS did not affect body weight in females, but GDX led to significant increases in body weight (Table 3), as expected [37]. GDX had no effect on adrenal weight in females (Table 3).

**Table 3:** SPS did not affect body weight or adrenal weight in females

Group		Body weight in grams (SEM)		Adrenal weight in grams (SEM)
		Baseline	Post-test	
Sham	Ctrl	199.4 (2.7)	213.0* (2.9)	0.34 (0.02)
*n* = 16/gp	SPS	199.4 (3.8)	209.4* (4.1)	0.37 (0.03)
GDX	Ctrl	215.7+ (4.3)	243.5*+ (4.5)	0.31 (0.01)
*n* = 15/gp	SPS	214.2+ (2.6)	234.5*+ (3.1)	0.30 (0.04)
GDX + T	Ctrl	227.0+ (4.9)	255.7*+ (5.9)	0.23 (0.03)
*n* = 11/gp	SPS	223.9+ (4.8)	246.2*+ (6.4)	0.27 (0.02)

Rat body weights were collected before undergoing SPS or control conditions (baseline) and before the dexamethasone suppression test (post-test). Adrenal weights were collected after sacrifice. Neither SPS nor testosterone (T) affected body or adrenal weight in females, and all rats gained weight over the course of the experiment (*, vs baseline). As expected, gonadectomy (GDX) increased female body weight (+, vs sham). Data was presented as mean±SEM. Significance set at *P* < .05 (indicated by asterisk) for planned pairwise comparisons (Bonferroni). Refer to Additional file 5 for full statistical results

## Discussion

Sex differences in the traumatic stress response of humans are among the most widely reported phenomena in epidemiological and clinical studies of PTSD, but the neurobiological basis for these differences is unknown, largely due to an overwhelming male bias in preclinical research [38]. We find robust and novel sex differences in the traumatic stress response of rats. Only males showed a hyper-responsive phenotype with an enhanced ASR and exaggerated negative feedback control of the stress hormone response, both core attributes of PTSD in men [5, 39]. Female rats, on the other hand, showed a unique phenotype with neither the ASR nor HPA negative feedback capturing the affective-like changes observed. Furthermore, these sex differences are independent of adult gonadal hormone status. These data have wide reaching implications for therapies to effectively treat PTSD in men and women.

Sex differences in the traumatic stress response were apparent at every level of analysis. For example, traumatic stress causes male rats to show an enhanced ASR while not affecting this measure in females. Moreover, negative feedback control of an acute stress response is enhanced by prior trauma in males, but unaffected or even decreased by prior trauma in females, suggesting that the female trauma response may share common attributes with depression, characterized by a blunted ASR [40] and reduced HPA negative feedback [10, 39]. Indeed, major depression is highly comorbid with PTSD, and also female-biased [6, 41, 42]. To explore this possibility, we added two common measures of depression, social interaction and sucrose preference. Each revealed female-specific responses to trauma, further supporting the view that the female response to traumatic stress is distinct from that of males and is reminiscent of depression. While overall, the effects of SPS in females point toward a depressive phenotype, the effects of SPS on sucrose preference was not consistent, indicating that this measure is only marginally sensitive to trauma in female rats. Thus, the traumatic stress response in females may not be equivalent to depression but is nonetheless, characterized by more affective dysfunction than that of males. These data align well with the clinical data on PTSD that women tend to show a more “internalizing” phenotype while men a more “externalizing” phenotype [3, 4, 24, 25]. Because other sex-biased psychiatric disorders share this same divide—externalizing versus internalizing symptomology—between men and women [43], our studies characterizing the underlying neuropathology of the traumatic stress response in males and females pave the way for gaining insight into the neurobiological underpinnings of other sex-biased psychiatric disorders. These diametrically opposed responses of male and female rats to trauma, while novel, conform well with the opposing effects of stress in males and females [23, 44–46].

Surprisingly, sex differences in the traumatic stress response were largely independent of adult circulating gonadal hormones. We find that the effects of traumatic stress on ASR and DEX suppression of CORT in males do not depend on adult circulating gonadal hormones, corroborating previous reports for ASR in males [47]. The null effect of SPS on these measures in females was also unaffected by gonadal hormones. While neither SPS nor gonadal hormones altered sucrose preference in males, both affected sucrose preference in females. Because ovariectomy did not alter sucrose preference in control females, the effect of trauma on sucrose preference in females appears to be mediated by adult ovarian hormones. This could mean that fluctuating estrogen levels influence the emergence and/or severity of depressive symptoms following exposure to trauma. However, whether ovarian hormones matter during and/or after experiencing trauma is entirely unanswered.

We found that SPS affected the latency to socially interact, a measure of time spent evaluating safety cues [48], but again, this effect was only seen in females. However, the effect of SPS on this measure in females also depended on housing conditions, with SPS decreasing the latency to socially interact when they were single-housed but *increasing* latency when pair-housed. We are the first to test this measure in a traumatic stress paradigm and demonstrate that the effects of stress on this measure extend to traumatic stress, with effects only in females. This same sex-specific effect was also seen in gonadectomized rats, indicating that the effect of SPS on this measure is independent of adult gonadal hormones. While SPS had no apparent effect on social interaction in gonadally intact males, castration did increase the latency of control males to socially interact. This, in turn, introduced an apparent effect of SPS to reduce social interaction latency (Fig. 3d). Whether this decreased latency reflects a genuine effect of SPS in castrates and hence, implicating androgens as protective against the deleterious effects of traumatic stress, or is an artifact of the effect of castration in unstressed males is unclear. Likewise, SPS increased GR expression in the PVN of intact males, but this effect was lost in castrated males because castration itself increased GR expression in the PVN of control males (Fig. 3c). Thus, castration may have protected GR expression from trauma in the PVN of males or may have simply masked the effect of trauma due to a ceiling effect. On the other hand, the effect of gonadectomy on the response to SPS in females is more straightforward. Ovariectomy eliminated the effect of SPS on GR expression in the PVN of females while not changing the number of GR+ neurons in the PVN of control, non-traumatized females. These data indicate that the effect of SPS on this measure, like sucrose preference, depends on ovarian hormones and raises questions about *when* gonadal hormones might be important—at the time of trauma and/or at the time of assessment. It is clear that differences in gonadal hormones change the response to acute stress. Thus, gonadal hormone status may also change the acute response to traumatic stress and ultimately whether the individual develops PTSD. In sum, while the effect of trauma on the ASR and DEX suppression of CORT appear independent of adult gonadal hormones, other measures like social interaction and sucrose preference seem to depend on hormonal status.

In the experiments reported here, we did not track estrous cycle stage in females because we first wanted to see if we could detect any sex differences in the traumatic stress response in a group of freely cycling females compared to males, which mirrors the case for the sex differences reported in the human PTSD literature. And indeed, we identified robust sex differences without regard to hormonal conditions. However, it is possible that some sex differences are only present during certain stages of the estrous cycle and were, thus, masked in a group of females selected from randomly cycling estrous stages, a circumstance that has been reported when investigating sex differences in fear extinction [49]. The results reported here indicate that ovarian hormones influence the female traumatic stress response with regard to sucrose preference and GR expression in the PVN. It is possible that these sex differences are driven by hormonal changes in the estrous cycle and exploring this possibility will offer more insight into within-sex individual differences but still would not change the fact that a sex difference in these measures exists when compared to males. The possibility that hormonal conditions during the time of trauma, during symptom assessment, and/or during treatment influence the traumatic stress response in different ways should be taken into account in future studies. Indeed, others have shown effects of ovarian hormone cycle stage on fear conditioning in healthy humans [50] and in female rats where these effects influence the efficacy of pharmacological interventions [51].

## Conclusions

Overall, adult gonadal hormones and housing conditions had little effect on the sex-specific responses to traumatic stress. This is particularly unexpected, given the current view that cycling estrogens robustly regulate stress susceptibility in females [52, 53]. Rather, it seems likely that these sex differences in the traumatic stress response are determined earlier in life, predisposing individuals to certain endophenotypes of PTSD as adults. This idea is consistent with reports that individual differences in the stress response are determined by weaning [54] and that neonatal hormones have a role in shaping the adult stress response in rats [55, 56]. The picture that emerges from our studies is not that female rats are more resilient to traumatic stress, as some have suggested [57], but that male and female rats show distinct phenotypes. Of note, measures of ASR and DST suggest that males are susceptible while females are resilient to traumatic stress, whereas measures of sucrose preference and social interaction suggest just the opposite—males are resilient and females are susceptible. Whether an individual is resilient or susceptible depends entirely on which outcome measure is being considered. In sum, the concept of resilience may not have heuristic value in the context of sex differences in the traumatic stress response.

We are the first to recapitulate distinct clinical subtypes of PTSD [3, 4, 58] linked to a single biological factor: sex. We propose that such adult sex differences reflect differences in the underlying neurobiology, orchestrated earlier in life by sex hormones and/or genes. Ultimately, by determining if and when gonadal hormones affect the traumatic stress response, which hormones are involved, which brain regions they act upon, and what changes the hormone(s) make in the brain regions mediating trauma responses, we can begin to map the neurobiology of those sex-specific responses to improve the diagnosis and treatment of PTSD.

## Additional files

Additional file 1: Effects of SPS on social interaction for females depend on housing condition. (a) SPS had no effect on the ASR in females, regardless of housing condition (single vs. paired) or when tested under bright lights. (b) Likewise, the DST was unaffected by SPS in females. Note however that while pair-housing increased the sensitivity to DEX, it did so for both SPS and control females. Thus, this effect is due to housing and not SPS. (c) SPS affected social interaction (based on latency to approach a novel female) but the direction of effect depended on the housing condition. SPS *decreased* the latency to approach when females were single-housed but *increased* the latency to approach when females were pair-housed. (d) SPS did not affect sucrose preference in females, regardless of housing condition, suggesting that the trauma response in females may not be a depressive-like phenotype. [Note however the observed effects on this measure only in females in the next study (Fig. 2).] (e) Neither SPS nor housing affected female body weight. These data, which replicate the null effect of SPS on ASR and DEX suppression, are consistent with the idea that the effects of traumatic stress for females are distinctly different from those of males and may share some traits of depression. Data are presented as mean ±SEM. Significance set at *P* < .05 (indicated by asterisk) for planned pairwise comparisons (Bonferroni). Refer to Additional file 2 for full statistical results. (DOCX 151 kb).

Additional file 2: Statistical results for data shown in Additional file 1 and Additional file 6. All pairwise comparisons use Bonferroni adjustment for multiple comparisons. RM denotes repeated measure, otherwise assume between group measures. Only statistically significant pairwise comparisons are shown. (DOCX 27 kb).

Additional file 3: Statistical results for data shown in Fig. 2, Table 1, and Additional file 6. All pairwise comparisons use Bonferroni adjustment for multiple comparisons. RM denotes repeated measure, otherwise assume between group measures. Only statistically significant pairwise comparisons are shown. (DOCX 27 kb).

Additional file 4: Statistical results for data shown in Fig. 3, Table 2, and Additional file 6. All pairwise comparisons use Bonferroni adjustment for multiple comparisons. RM denotes repeated measure, otherwise assume between group measures. Only statistically significant pairwise comparisons are shown. (DOCX 40 kb).

Additional file 5: Statistical results for data shown in Fig. 4, Table 3, and Additional file 6. All pairwise comparisons use Bonferroni adjustment for multiple comparisons. RM denotes repeated measure. otherwise assume between group measures. Only statistically significant pairwise comparisons are shown. (DOCX 30 kb).

Additional file 6: Social interaction measures of latency to approach the empty interaction zone and total time spent in social interaction zone. (a) Neither housing nor SPS affected the latency of females to approach the empty rat enclosure, indicating that the effect of SPS is on social interaction per se and not on general activity or exploration of the chamber. (b) Female rats in all groups showed a significant increase in total time spent in the interaction zone when a novel rat was present, regardless of traumatic stress exposure or housing. (c) Sham-operated (intact) control females approached the empty interaction zone more quickly than males, and this effect was independent of SPS. (d) All sham-operated rats spent more time in the interaction zone when a novel rat was present regardless of traumatic stress exposure or sex. (e) SPS had no effect on latency to enter the empty interaction zone, regardless of gonadal status, but gonadectomy (GDX) reduced this latency measure independent of SPS, an effect reversed by T replacement, suggesting that T normally affects overall activity levels in males. (f) All male rats spent more time in the interaction zone when a novel rat was present regardless of traumatic stress exposure or hormone status. (g) Only the combination of SPS and T treatment significantly increased the latency to enter the empty interaction zone in females. (f) All female rats spent more time in the interaction zone when a novel rat was present, regardless of traumatic stress exposure or hormone status. Data presented as mean±SEM. Significance set at *P* < .05 (*) for planned pairwise comparisons (Bonferroni). See Additional files 2, 3, 4, and 5 for full statistics. (DOCX 109 kb).

## Abbreviations

ASR: Acoustic startle response
CORT: Corticosterone
DEX: Dexamethasone
DST: Dexamethasone suppression test
GDX: Gonadecomty/gonadectomized
GR: Glucocorticoid receptor
HPA: Hypothalamic-pituitary-adrenal
IHC: Immunohistochemistry
PTSD: Post-traumatic stress disorder
PVN: Paraventricular nucleus of the hypothalamus
SPS: Single prolonged stress
T: Testosterone
