# Supplementary material for: Sex differences in the traumatic stress response: the role of adult gonadal hormones
Source: Biol Sex Differ. 2018 Jul 13;9:32. doi: 10.1186/s13293-018-0192-8 (PMC6043950; doi:10.1186/s13293-018-0192-8)
Supplement: Supplementary file 1 — Effects of SPS on social interaction for females depend on housing condition. (a) SPS had no effect on the ASR in females, regardless of housing condition (single vs. paired) or when tested under bright lights. (b) Likewise, the DST was unaffected by SPS in females. Note however that while pair-housing increased the sensitivity to DEX, it did so for both SPS and control females. Thus, this effect is due to housing and not SPS. (c) SPS affected social interaction (based on latency to approach a novel female) but the direction of effect depended on the housing condition. SPS decreased the latency to approach when females were single-housed but increased the latency to approach when females were pair-housed. (d) SPS did not affect sucrose preference in females, regardless of housing condition, suggesting that the trauma response in females may not be a depressive-like phenotype. [Note however the observed effects on this measure only in females in the next study (Fig. 2).] (e) Neither SPS nor housing affected female body weight. These data, which replicate the null effect of SPS on ASR and DEX suppression, are consistent with the idea that the effects of traumatic stress for females are distinctly different from those of males and may share some traits of depression. Data are presented as mean ±SEM. Significance set at P < .05 (indicated by asterisk) for planned pairwise comparisons (Bonferroni). Refer to Additional file 2 for full statistical results. (DOCX 151 kb). [file 13293_2018_192_MOESM1_ESM.docx]

**a b**

**c d e**


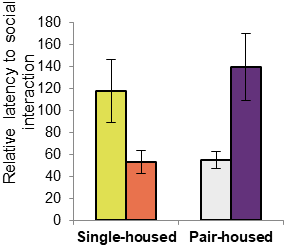


*

*

*


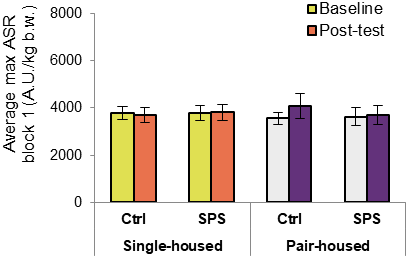

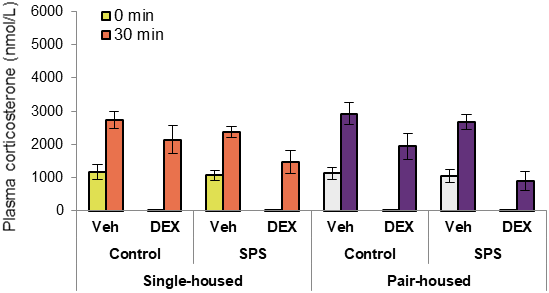


*

*

*

*

*

*

*

*

*

*


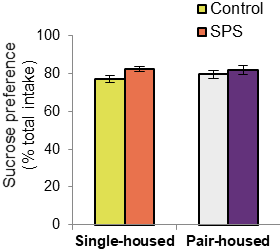


**DEX suppression test**

**Acoustic startle**

12

11

10

11

11

12

12

10

16

16

17

17

**Body weight**

**Sucrose preference**

**Social interaction**

n=24/gp


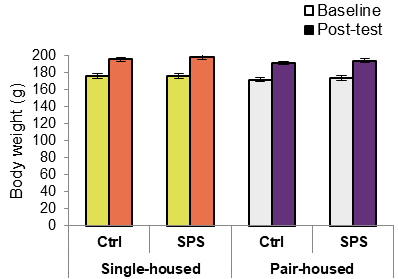


*

*

*

*

22

21

20

21

22

23

24

21
