# Supplementary material for: Sex differences in the traumatic stress response: the role of adult gonadal hormones
Source: Biol Sex Differ. 2018 Jul 13;9:32. doi: 10.1186/s13293-018-0192-8 (PMC6043950; doi:10.1186/s13293-018-0192-8)
Supplement: Supplementary file 2 — Statistical results for data shown in Additional file 1 and Additional file 6. All pairwise comparisons use Bonferroni adjustment for multiple comparisons. RM denotes repeated measure, otherwise assume between group measures. Only statistically significant pairwise comparisons are shown. (DOCX 27 kb). [file 13293_2018_192_MOESM2_ESM.docx]

| **Additional file 2. Statistical readouts for data shown in Additional file 1** **and Additional file 6. All pairwise comparisons use Bonferroni adjustment for multiple comparisons. RM denotes repeated measure; otherwise assume between group measures. Only statistically significant pairwise comparisons are shown.** | | | | |  |
| --- | --- | --- | --- | --- | --- |
| *Outcome measure* | *Statistical test* | *Significant effects* | *p value* | *Power (α=0.05)* | *Partial eta^2^* |
| **ASR (Addl file 1a)** | RM 3-way ANOVA (stress*housing*time) | Main effect: time (*F*­_1,62_=0.393) | 0.533 | 0.095 | 0.006 |
|  |  | Main effect: stress (*F*­_1,62_=0.019) | 0.891 | 0.052 | 0.000 |
|  |  | Main effect: housing (*F*­_1,62_=0.008) | 0.928 | 0.051 | 0.000 |
|  |  | Interaction: time*stress (*F*­_1,62_=0.150) | 0.700 | 0.067 | 0.002 |
|  |  | Interaction: time *housing (*F*­_1,62_=0.602) | 0.441 | 0.119 | 0.010 |
|  |  | Interaction: stress*housing (*F*­_1,62_=0.131) | 0.719 | 0.065 | 0.002 |
|  |  | Int: stress*housing*time (*F*­_1,62_=0.395) | 0.532 | 0.095 | 0.006 |
| **DST (Addl file 1b)** | RM 4-way ANOVA (stress*housing*time*DEX) | Main effect: time (*F*­_1,81_=188.820) | <0.0001 | 1.000 | 0.700 |
|  |  | Main effect: stress (*F*­_1,81_=6.502) | 0.013 | 0.712 | 0.074 |
|  |  | Main effect: DEX (*F*­_1,81_=74.502) | <0.0001 | 1.000 | 0.479 |
|  |  | Main effect: housing (*F*­_1,81_=0.101) | 0.752 | 0.061 | 0.001 |
|  |  | Interaction: time*housing (*F*­_1,81_=0.076) | 0.784 | 0.059 | 0.001 |
|  |  | Interaction: time*stress (*F*­_1,81_=5.299) | 0.024 | 0.623 | 0.061 |
|  |  | Interaction: time*DEX (*F*­_1,81_=0.005) | 0.946 | 0.051 | 0.000 |
|  |  | Int: time*housing*stress (*F*­_1,81_=0.124) | 0.726 | 0.064 | 0.002 |
|  |  | Int: time*housing*DEX (*F*­_1,81_=2.215) | 0.141 | 0.313 | 0.027 |
|  |  | Int: time*stress*DEX (*F*­_1,81_=1.899) | 0.172 | 0.275 | 0.023 |
|  |  | Int: time*housing*stress*DEX  (*F*­_1,81_=0.258) | 0.613 | 0.079 | 0.003 |
|  |  | Interaction: housing*stress (*F*­_1,81_=0.051) | 0.822 | 0.056 | 0.001 |
|  |  | Interaction: housing*DEX (*F*­_1,81_=1.500) | 0.224 | 0.227 | 0.018 |
|  |  | Interaction: stress*DEX (*F*­_1,81_=0.892) | 0.348 | 0.154 | 0.011 |
|  |  | Int: housing*stress*DEX (*F*­_1,81_=0.282) | 0.597 | 0.082 | 0.003 |
| *Because there was no effect of housing on DST, a separate 3-way ANOVA was run for single- and pair-housed females* | | | | | |
| **DST, single-housed (Addl file 1b)** | RM 3-way ANOVA (stress*time*DEX) | Main effect: time (*F*­_1,41_=95.149) | <0.0001 | 1.000 | 0.699 |
|  |  | Main effect: DEX (*F*­_1,41_=26.732) | <0.0001 | 0.999 | 0.395 |
|  |  | Main effect: stress (*F*­_1,41_=2.631) | 0.112 | 0.354 | 0.060 |
|  |  | Interaction: time*stress (*F*­_1,41_=1.842) | 0.182 | 0.263 | 0.043 |
|  |  | Interaction: time*DEX (*F*­_1,41_=1.172) | 0.285 | 0.185 | 0.028 |
|  |  | Int.: time*stress*DEX (*F*­_1,41_=0.367) | 0.548 | 0.091 | 0.009 |
|  |  | Interaction: stress*DEX (*F*­_1,41_=0.083) | 0.774 | 0.059 | 0.002 |
|  |  | Pairwise: SPS time 1 DEX v. veh | <0.0001 | 0.999 |  |
|  |  | Pairwise: control time 1 DEX v. veh | <0.0001 | 1.000 |  |
|  |  | Pairwise: SPS DEX time 1 v. 2 | <0.0001 | 0.984 |  |
|  |  | Pairwise: SPS vehicle time 1 v. 2 | <0.0001 | 0.980 |  |
|  |  | Pairwise: control DEX time 1 v. 2 | <0.0001 | 1.000 |  |
|  |  | Pairwise: control vehicle time 1 v. 2 | <0.0001 | 0.995 |  |
| **DST, pair-housed (Addl file 1b)** | RM 3-way ANOVA (stress*time*DEX) | Main effect: time (*F*­_1,40_=93.895) | <0.0001 | 1.000 | 0.701 |
|  |  | Main effect: DEX (*F*­_1,40_=49.963) | <0.0001 | 1.000 | 0.555 |
|  |  | Main effect: stress (*F*­_1,40_=3.963) | 0.053 | 0.493 | 0.090 |
|  |  | Interaction: time*stress (*F*­_1,40_=3.647) | 0.063 | 0.462 | 0.084 |
|  |  | Interaction: time*DEX (*F*­_1,40_=1.045) | 0.313 | 0.170 | 0.025 |
|  |  | Int.: time*stress*DEX (*F*­_1,40_=1.841) | 0.182 | 0.263 | 0.044 |
|  |  | Interaction: stress*DEX (*F*­_1,40_=1.120) | 0.293 | 0.178 | 0.027 |
|  |  | Pairwise: DEX time 2 SPS v. control | 0.020 | 0.654 |  |
|  |  | Pairwise: SPS time 1 DEX v. veh | <0.0001 | 1.000 |  |
|  |  | Pairwise: SPS time 2 DEX v. veh | <0.0001 | 0.984 |  |
|  |  | Pairwise: control time 1 DEX v. veh | <0.0001 | 1.000 |  |
|  |  | Pairwise: control time 2 DEX v. veh | 0.032 | 0.585 |  |
|  |  | Pairwise: SPS DEX time 1 v. 2 | 0.007 | 0.788 |  |
|  |  | Pairwise: SPS vehicle time 1 v. 2 | <0.0001 | 0.999 |  |
|  |  | Pairwise: control DEX time 1 v. 2 | <0.0001 | 1.000 |  |
|  |  | Pairwise: control vehicle time 1 v. 2 | <0.0001 | 1.000 |  |
| **Sucrose pref. (Addl file 1d)** | 2-way ANOVA (stress*housing) | Main effect: stress (*F*­_1,80_=3.616) | 0.061 | 0.468 |  |
|  |  | Main effect: housing (*F*­_1,80_=0.185) | 0.668 | 0.071 |  |
|  |  | Interaction: stress*housing (*F*­_1,80_=0.575) | 0.451 | 0.116 |  |
| **Social interaction (Addl file 1c)** | 2-way ANOVA (stress*housing) | Main effect: stress (*F*­_1,86_=0.193) | 0.662 | 0.072 | 0.002 |
|  |  | Main effect: housing (*F*­_1,86_=0.267) | 0.607 | 0.080 | 0.003 |
|  |  | Int.: stress*housing (*F*­_1,86_=10.835) | 0.001 | 0.902 | 0.112 |
|  |  | Pairwise: pair-housed SPS v. control | 0.009 | 0.751 |  |
|  |  | Pairwise: single-housed SPS v. control | 0.049 | 0.505 |  |
|  |  | Pairwise: SPS pair-housed v. single-housed | 0.009 | 0.758 |  |
| **Latency/empty zone (Add. file 6a)** | 2-way ANOVA (stress*housing) | Main effect: stress (*F*­_1,86_=0.047) | 0.828 | 0.055 | 0.001 |
|  |  | Main effect: housing (*F*­_1,86_=1.184) | 0.280 | 0.190 | 0.014 |
|  |  | Int.: stress*housing (*F*­_1,86_=3.145) | 0.080 | 0.418 | 0.035 |
| **Total social int. (Add. file 6b)** | RM 3-way ANOVA (stress*housing*target) | Main effect: target (*F*­_1,86_=259.136) | <0.0001 | 1.000 | 0.751 |
|  |  | Main effect: stress (*F*­_1,86_=0.007) | 0.933 | 0.051 | 0.000 |
|  |  | Main effect: housing (*F*­_1,86_=2.648) | 0.107 | 0.363 | 0.030 |
|  |  | Interaction: target*stress (*F*­_1,86_=0.196) | 0.659 | 0.072 | 0.002 |
|  |  | Interaction: target*housing (*F*­_1,86_=0.468) | 0.496 | 0.104 | 0.005 |
|  |  | Int.: target*housing*stress (*F*­_1,86_=0.046) | 0.830 | 0.055 | 0.001 |
|  |  | Interaction stress*housing (*F*­_1,86_=0.338) | 0.563 | 0.089 | 0.004 |
|  |  | Pairwise: SPS single target yes v. no | <0.0001 | 1.000 |  |
|  |  | Pairwise: Control single target yes v. no | <0.0001 | 1.000 |  |
|  |  | Pairwise: SPS pair target yes v. no | <0.0001 | 1.000 |  |
|  |  | Pairwise: Control pair target yes v. no | <0.0001 | 1.000 |  |
| **Body wt.**  **(Addl file 1e)** | RM 3-way ANOVA (stress*housing*time) | Main effect: time (*F*­_1,92_=646.656) | <0.0001 | 1.000 | 0.875 |
|  |  | Main effect: stress (*F*­_1,92_=0.514) | 0.475 | 0.109 | 0.006 |
|  |  | Main effect: housing (*F*­_1,92_=2.324) | 0.131 | 0.326 | 0.025 |
|  |  | Interaction: time *stress (*F*­_1,92_=2.143) | 0.147 | 0.305 | 0.023 |
|  |  | Interaction: time*housing (*F*­_1,92_=0.436) | 0.510 | 0.100 | 0.005 |
|  |  | Int.: time*stress*housing (*F*­_1,92_=0.403) | 0.527 | 0.096 | 0.004 |
|  |  | Interaction: stress*housing (*F*­_1,92_=0.043) | 0.836 | 0.055 | 0.000 |
|  |  | Pairwise: SPS single time 1 v. 2 | <0.0001 | 1.000 |  |
|  |  | Pairwise: Control single time 1 v. 2 | <0.0001 | 1.000 |  |
|  |  | Pairwise: SPS pair time 1 v. 2 | <0.0001 | 1.000 |  |
|  |  | Pairwise: Control pair time 1 v. 2 | <0.0001 | 1.000 |  |
