# Supplementary material for: Sex differences in the traumatic stress response: the role of adult gonadal hormones
Source: Biol Sex Differ. 2018 Jul 13;9:32. doi: 10.1186/s13293-018-0192-8 (PMC6043950; doi:10.1186/s13293-018-0192-8)
Supplement: Supplementary file 3 — Statistical results for data shown in Fig. 2, Table 1, and Additional file 6. All pairwise comparisons use Bonferroni adjustment for multiple comparisons. RM denotes repeated measure, otherwise assume between group measures. Only statistically significant pairwise comparisons are shown. (DOCX 27 kb). [file 13293_2018_192_MOESM3_ESM.docx]

| **Additional file 3. Statistical results for data shown in Figure 2, Table 1, and Additional file 6. All pairwise comparisons use Bonferroni adjustment for multiple comparisons. RM denotes repeated measure; otherwise assume between group measures. Only statistically significant pairwise comparisons are shown.** | | | | |  |
| --- | --- | --- | --- | --- | --- |
| *Outcome measure* | *Statistical test* | *Significant effects* | *p value* | *Power (α=0.05)* | *Partial eta^2^* |
| **ASR**  **(Fig. 2a)** | RM 3-way ANOVA (stress*sex*time) | Main effect: time (*F*­_1,56_=5.762) | 0.020 | 0.655 | 0.093 |
|  |  | Main effect: stress (*F*­_1,56_=0.949) | 0.334 | 0.160 | 0.017 |
|  |  | Main effect: sex (*F*­_1,56_=0.010) | 0.920 | 0.051 | 0.000 |
|  |  | Interaction: stress*sex (*F*­_1,56_=0.835) | 0.365 | 0.146 | 0.015 |
|  |  | Interaction: stress*time (*F*­_1,56_=7.494) | 0.008 | 0.767 | 0.118 |
|  |  | Interaction: sex*time (*F*­_1,56_=4.862) | 0.032 | 0.582 | 0.080 |
|  |  | Interaction: stress*sex*time (*F*­_1,56_=5.550) | 0.022 | 0.639 | 0.090 |
|  |  | Pairwise: SPS male time 1 v. 2 | 0.008 | 0.767 |  |
|  |  | Pairwise: SPS female time 1 v. 2 | 0.019 | 0.660 |  |
|  |  | Pairwise: control male time 1 v. 2 | 0.021 | 0.644 |  |
|  |  | Pairwise: control female time 1 v. 2 | 0.033 | 0.575 |  |
| **DST**  **(Fig. 2b)** | RM 4-way ANOVA (stress*sex*time*DEX) | Main effect: time (*F*­_1,54_=143.423) | <0.0001 | 1.000 | 0.726 |
|  |  | Main effect: DEX (*F*­_1,54_=102.205) | <0.0001 | 1.000 | 0.654 |
|  |  | Main effect: sex (*F*­_1,54_=89.564) | <0.0001 | 1.000 | 0.624 |
|  |  | Main effect: stress (*F*­_1,54_=0.291) | 0.592 | 0.083 | 0.005 |
|  |  | Interaction: time*DEX (*F*­_1,54_=3.033) | 0.087 | 0.402 | 0.053 |
|  |  | Interaction: time*stress*DEX (*F*­_1,54_=0.300) | 0.586 | 0.084 | 0.006 |
|  |  | Interaction: time*stress*sex (*F*­_1,54_=1.251) | 0.268 | 0.196 | 0.023 |
|  |  | Interaction: time*DEX*sex (*F*­_1,54_=2.465) | 0.122 | 0.338 | 0.044 |
|  |  | Interaction: time*DEX*sex*stress (*F*­_1,54_=0.716) | 0.401 | 0.132 | 0.013 |
|  |  | Interaction: stress*DEX (*F*­_1,54_=2.422) | 0.125 | 0.333 | 0.043 |
|  |  | Interaction: stress*sex (*F*­_1,54_=0.027) | 0.869 | 0.053 | 0.001 |
|  |  | Interaction: stress*DEX*sex (*F*­_1,54_=0.030) | 0.863 | 0.053 | 0.001 |
|  |  | Interaction: stress*time (*F*­_1,54_=8.439) | 0.005 | 0.814 | 0.135 |
|  |  | Interaction: sex*time (*F*­_1,54_=26.272) | <0.0001 | 0.999 | 0.327 |
|  |  | Interaction: sex*DEX (*F*­_1,54_=9.059) | 0.004 | 0.840 | 0.144 |
|  |  | Pairwise: veh female time 1 SPS v. control | <0.0001 | 0.969 |  |
|  |  | Pairwise: SPS male time 1 DEX v. veh | 0.002 | 0.887 |  |
|  |  | Pairwise: SPS male time 2 DEX v. veh | 0.001 | 0.914 |  |
|  |  | Pairwise: SPS female time 1 DEX v. veh | <0.0001 | 1.000 |  |
|  |  | Pairwise: SPS female time 2 DEX v. veh | 0.001 | 0.950 |  |
|  |  | Pairwise: control male time 1 DEX v. veh | 0.048 | 0.512 |  |
|  |  | Pairwise: control male time 2 DEX v. veh | 0.010 | 0.746 |  |
|  |  | Pairwise: control female time 1 DEX v. veh | <0.0001 | 1.000 |  |
|  |  | Pairwise: control female time 2 DEX v. veh | 0.001 | 0.951 |  |
|  |  | Pairwise: SPS DEX time 2 male v. female | 0.003 | 0.875 |  |
|  |  | Pairwise: SPS vehicle time 1 male v. female | <0.0001 | 1.000 |  |
|  |  | Pairwise: SPS vehicle time 2 male v. female | <0.0001 | 0.988 |  |
|  |  | Pairwise: control DEX time 2 male v. female | <0.0001 | 0.960 |  |
|  |  | Pairwise: control vehicle time 1 male v. female | <0.0001 | 0.996 |  |
|  |  | Pairwise: control vehicle time 2 male v. female | <0.0001 | 0.999 |  |
|  |  | Pairwise: SPS DEX female time 1 v. 2 | <0.0001 | 0.993 |  |
|  |  | Pairwise: SPS vehicle male time 1 v. 2 | 0.001 | 0.935 |  |
|  |  | Pairwise: SPS vehicle female time 1 v. 2 | <0.0001 | 0.978 |  |
|  |  | Pairwise: control DEX male time 1 v. 2 | 0.034 | 0.571 |  |
|  |  | Pairwise: control DEX female time 1 v. 2 | <0.0001 | 1.000 |  |
|  |  | Pairwise: control vehicle male time 1 v. 2 | <0.0001 | 0.993 |  |
|  |  | Pairwise: control vehicle female time 1 v. 2 | <0.0001 | 1.000 |  |
| **Adrenal wt (Table 1)** | 2-way ANOVA (stress*sex) | Main effect: sex (*F*­_1,23_=68.151) | <0.0001 | 1.000 | 0.773 |
|  |  | Main effect: stress (*F*­_1,23_=0.203) | 0.657 | 0.071 | 0.010 |
|  |  | Interaction: stress*sex (*F*­_1,23_=1.166) | 0.293 | 0.177 | 0.055 |
|  |  | Pairwise: SPS male v. female | <0.0001 | 1.000 |  |
|  |  | Pairwise: control male v. female | <0.0001 | 0.998 |  |
| **PVN GR**  **(Fig. 2c)** | 2-way ANOVA (stress*sex) | Main effect: stress (*F*­_1,16_=0.118) | 0.736 | 0.062 | 0.007 |
|  |  | Main effect: sex (*F*­_1,16_=2.695) | 0.120 | 0.339 | 0.144 |
|  |  | Interaction: stress*sex (*F*­_1,16_=10.784) | 0.005 | 0.869 | 0.403 |
|  |  | Pairwise: control male v. female | 0.004 | 0.876 |  |
|  |  | Pairwise: male SPS v. control | 0.020 | 0.490 |  |
|  |  | Pairwise: female SPS v. control | 0.056 | 0.682 |  |
| **Sucrose pref. (Fig. 2e)** | 2-way ANOVA (stress*sex) | Main effect: stress (*F*­_1,48_=0.385) | 0.538 | 0.093 | 0.008 |
|  |  | Main effect: sex (*F*­_1,48_=1.183) | 0.282 | 0.187 | 0.024 |
|  |  | Interaction: stress*sex (*F*­_1,48_=6.410) | 0.015 | 0.699 | 0.118 |
|  |  | Pairwise: female SPS v. control | 0.034 | 0.570 |  |
|  |  | Pairwise: SPS male v. female | 0.017 | 0.675 |  |
| **Social interaction (Fig. 2d)** | 2-way ANOVA (stress*sex) | Main effect: stress (*F*­_1,54_=0.938) | 0.337 | 0.158 | 0.017 |
|  |  | Main effect: sex (*F*­_1,54_=3.599) | 0.063 | 0.462 | 0.062 |
|  |  | Interaction: stress*sex (*F*­_1,54_=5.467) | 0.023 | 0.632 | 0.092 |
|  |  | Pairwise: female SPS v. control | 0.023 | 0.632 |  |
|  |  | Pairwise: control female v. male | 0.004 | 0.849 |  |
| **Latency/empty zone**  **(Add. file 6c)** | 2-way ANOVA (stress*sex) | Main effect: sex (*F*­_1,54_=6.596) | 0.011 | 0.736 | 0.114 |
|  |  | Main effect: stress (*F*­_1,54_=0.068) | 0.795 | 0.058 | 0.001 |
|  |  | Interaction: sex*stress (*F*­_1,54_=0.408) | 0.526 | 0.096 | 0.007 |
|  |  | Pairwise: control male v. female | 0.022 | 0.639 |  |
| **Body weight (Table 1)** | RM 3-way ANOVA (stress*sex*time) | Main effect: sex (*F*­_1,60_=487.320) | <0.0001 | 1.000 | 0.890 |
|  |  | Main effect: time (*F*­_1,60_=184.248) | <0.0001 | 1.000 | 0.754 |
|  |  | Main effect: stress (*F*­_1,60_=0.123) | 0.727 | 0.064 | 0.002 |
|  |  | Interaction: time*stress (*F*­_1,60_=0.371) | 0.545 | 0.092 | 0.006 |
|  |  | Interaction: time*stress*sex (*F*­_1,60_=0.346) | 0.559 | 0.089 | 0.006 |
|  |  | Interaction: sex*stress (*F*­_1,60_=0.493) | 0.485 | 0.106 | 0.008 |
|  |  | Interaction: time*sex (*F*­_1,60_=31.948) | <0.0001 | 1.000 | 0.347 |
|  |  | Pairwise: SPS time 1 male v. female | <0.0001 | 1.000 |  |
|  |  | Pairwise: SPS time 2 male v. female | <0.0001 | 1.000 |  |
|  |  | Pairwise: Control time 1 male v. female | <0.0001 | 1.000 |  |
|  |  | Pairwise: Control time 2 male v. female | <0.0001 | 1.000 |  |
|  |  | Pairwise: SPS male time 1 v. 2 | <0.0001 | 1.000 |  |
|  |  | Pairwise: Control male time 1 v. 2 | <0.0001 | 1.000 |  |
|  |  | Pairwise: SPS female time 1 v. 2 | <0.0001 | 0.911 |  |
|  |  | Pairwise: Control female time 1 v. 2 | <0.0001 | 0.994 |  |
| **Total social int. (Add. file 6d)** | RM 3-way ANOVA (stress*sex*target) | Main effect: target (*F*­_1,54_=83.119) | <0.0001 | 1.000 | 0.606 |
|  |  | Main effect: sex (*F*­_1,54_=1.785) | 0.187 | 0.259 | 0.032 |
|  |  | Main effect: stress (*F*­_1,54_=1.270) | 0.265 | 0.198 | 0.023 |
|  |  | Interaction: target*stress (*F*­_1,54_=0.856) | 0.359 | 0.149 | 0.016 |
|  |  | Interaction: target*sex (*F*­_1,54_=0.276) | 0.602 | 0.081 | 0.005 |
|  |  | Interaction: target*stress*sex (*F*­_1,54_=0.789) | 0.378 | 0.141 | 0.014 |
|  |  | Interaction: stress*sex (*F*­_1,54_=3.551) | 0.065 | 0.457 | 0.062 |
|  |  | Pairwise: SPS male target yes vs. no | <0.0001 | 1.000 |  |
|  |  | Pairwise: Control male target yes vs. no | <0.0001 | 1.000 |  |
|  |  | Pairwise: SPS female target yes vs. no | <0.0001 | 1.000 |  |
|  |  | Pairwise: Control female target yes vs. no | <0.0001 | 1.000 |  |
