# Supplementary material for: Sex differences in the traumatic stress response: the role of adult gonadal hormones
Source: Biol Sex Differ. 2018 Jul 13;9:32. doi: 10.1186/s13293-018-0192-8 (PMC6043950; doi:10.1186/s13293-018-0192-8)
Supplement: Supplementary file 5 — Statistical results for data shown in Fig. 4, Table 3, and Additional file 6. All pairwise comparisons use Bonferroni adjustment for multiple comparisons. RM denotes repeated measure. otherwise assume between group measures. Only statistically significant pairwise comparisons are shown. (DOCX 30 kb). [file 13293_2018_192_MOESM5_ESM.docx]

| **Additional file 5. Statistical results for data shown in Figure 4, Table 3, and Additional file 6. All pairwise comparisons use Bonferroni adjustment for multiple comparisons. RM denotes repeated measure; otherwise assume between group measures. Only statistically significant pairwise comparisons are shown.** | | | | |  |
| --- | --- | --- | --- | --- | --- |
| *Outcome measure* | *Statistical test* | *Significant effects* | *p value* | *Power (α=0.05)* | *Partial eta^2^* |
| **ASR (Fig. 4a)** | RM 3-way ANOVA (stress*GDX*time) sham vs GDX | Main effect: time (*F*­_1,55_=15.542) | <0.0001 | 0.972 | 0.220 |
|  |  | Main effect: stress (*F*­_1,55_=1.054) | 0.309 | 0.172 | 0.019 |
|  |  | Main effect: GDX (*F*­_1,55_=1.152) | 0.288 | 0.184 | 0.021 |
|  |  | Interaction: time*stress (*F*­_1,55_=0.384) | 0.538 | 0.093 | 0.007 |
|  |  | Interaction: time*GDX (*F*­_1,55_=0.200) | 0.656 | 0.072 | 0.004 |
|  |  | Interaction: stress*GDX (*F*­_1,55_=0.545) | 0.484 | 0.112 | 0.010 |
|  |  | Interaction: time*stress*GDX (*F*­_1,55_=0.967) | 0.330 | 0.162 | 0.017 |
|  |  | Pairwise: SPS sham time 1 v. 2 | 0.024 | 0.625 |  |
|  |  | Pairwise: Control sham time 1 v. 2 | 0.040 | 0.541 |  |
|  |  | Pairwise: Control GDX time 1 v. 2 | 0.013 | 0.715 |  |
|  | RM 3-way ANOVA (stress*GDX*time)  GDX v. GDX+T | Main effect: time (*F*­_1,47_=6.509) | <0.0001 | 0.705 | 0.122 |
|  |  | Main effect: stress (*F*­_1,47_=0.166) | 0.686 | 0.068 | 0.004 |
|  |  | Main effect: GDX (*F*­_1,47_=0.031) | 0.860 | 0.053 | 0.001 |
|  |  | Interaction: time*stress (*F*­_1,47_=2.574) | 0.115 | 0.349 | 0.115 |
|  |  | Interaction: time*GDX (*F*­_1,47_=0.017) | 0.896 | 0.052 | 0.000 |
|  |  | Interaction: stress*GDX (*F*­_1,47_=0.026) | 0.872 | 0.053 | 0.001 |
|  |  | Interaction: time*stress*GDX (*F*­_1,47_=0.140) | 0.710 | 0.066 | 0.003 |
|  |  | Pairwise: Control GDX time 1 v. 2 | 0.037 | 0.555 |  |
|  |  | Pairwise: Control GDX+T time 1 v. 2 | 0.045 | 0.524 |  |
| **DST (Fig. 4b)** | RM 4-way ANOVA (stress*GDX*time*DEX) sham v. GDX | Main effect: time (*F*­_1,48_=182.650) | <0.0001 | 1.000 | 0.792 |
|  |  | Main effect: DEX (*F*­_1,48_=94.902) | <0.0001 | 1.000 | 0.664 |
|  |  | Interaction: stress*time*GDX (*F*­_1,48_=4.266) | 0.044 | 0.526 | 0.082 |
|  |  | Main effect: stress (*F*­_1,48_=0.300) | 0.586 | 0.084 | 0.006 |
|  |  | Main effect: GDX (*F*­_1,48_=2.836) | 0.099 | 0.379 | 0.056 |
|  |  | Interaction: stress*GDX (*F*­_1,48_=0.769) | 0.385 | 0.138 | 0.016 |
|  |  | Interaction: stress*DEX (*F*­_1,48_=2.447) | 0.124 | 0.335 | 0.049 |
|  |  | Interaction: GDX*DEX (*F*­_1,48_=3.216) | 0.079 | 0.420 | 0.063 |
|  |  | Interaction: stress*GDX*DEX (*F*­_1,48_=0.002) | 0.966 | 0.050 | 0.000 |
|  |  | Interaction: stress*time (*F*­_1,48_=1.977) | 0.166 | 0.281 | 0.040 |
|  |  | Interaction: DEX*time (*F*­_1,48_=0.982) | 0.327 | 0.163 | 0.020 |
|  |  | Interaction: time*GDX (*F*­_1,48_=1.538) | 0.221 | 0.229 | 0.031 |
|  |  | Interaction: time*stress*DEX (*F*­_1,48_=0.097) | 0.756 | 0.061 | 0.002 |
|  |  | Interaction: time*GDX*DEX (*F*­_1,48_=1.297) | 0.260 | 0.200 | 0.026 |
|  |  | Int.: time*stress*GDX*DEX (*F*­_1,48_=0.793) | 0.378 | 0.141 | 0.016 |
|  |  | Pairwise: vehicle sham time 1 SPS v. control | <0.0001 | 0.962 |  |
|  |  | Pairwise: SPS sham time 1 DEX v. veh | <0.0001 | 1.000 |  |
|  |  | Pairwise: SPS sham time 2 DEX v. veh | 0.002 | 0.897 |  |
|  |  | Pairwise: SPS GDX time 1 DEX v. veh | <0.0001 | 1.000 |  |
|  |  | Pairwise: SPS GDX time 2 DEX v. veh | 0.027 | 0.608 |  |
|  |  | Pairwise: control sham time 1 DEX v. veh | <0.0001 | 1.000 |  |
|  |  | Pairwise: control sham time 2 DEX v. veh | 0.002 | 0.899 |  |
|  |  | Pairwise: control GDX time 1 DEX v. veh | <0.0001 | 1.000 |  |
|  |  | Pairwise: SPS vehicle time 1 sham v. GDX | 0.038 | 0.550 |  |
|  |  | Pairwise: control vehicle time 2 sham v. GDX | 0.012 | 0.730 |  |
|  |  | Pairwise: SPS DEX sham time 1 v. time 2 | <0.0001 | 0.979 |  |
|  |  | Pairwise: SPS DEX GDX time 1 v. time 2 | <0.0001 | 0.997 |  |
|  |  | Pairwise: SPS vehicle sham time 1 v. time 2 | 0.001 | 0.946 |  |
|  |  | Pairwise: SPS vehicle GDX time 1 v. time 2 | <0.0001 | 0.989 |  |
|  |  | Pairwise: control DEX sham time 1 v. time 2 | <0.0001 | 1.000 |  |
|  |  | Pairwise: control DEX GDX time 1 v. time 2 | <0.0001 | 1.000 |  |
|  |  | Pairwise: control vehicle sham time 1 v. time 2 | <0.0001 | 1.000 |  |
|  |  | Pairwise: control vehicle GDX time 1 v. time 2 | 0.005 | 0.821 |  |
|  | RM 4-way ANOVA (stress*GDX*time*DEX) GDX vs GDX+T | Main effect: time (*F*­_1,40_=178.150) | <0.0001 | 1.000 | 0.817 |
|  |  | Main effect: DEX (*F*­_1,40_=38.089) | <0.0001 | 1.000 | 0.488 |
|  |  | Main effect: GDX (*F*­_1,40_=12.186) | 0.001 | 0.926 | 0.234 |
|  |  | Interaction: time*DEX*GDX (*F*­_1,40_=4.441) | 0.041 | 0.539 | 0.100 |
|  |  | Interaction: stress*time*GDX (*F*­_1,40_=0.369) | 0.547 | 0.091 | 0.009 |
|  |  | Main effect: stress (*F*­_1,40_=0.470) | 0.497 | 0.103 | 0.012 |
|  |  | Interaction: stress*GDX (*F*­_1,40_=0.446) | 0.508 | 0.100 | 0.011 |
|  |  | Interaction: stress*DEX (*F*­_1,40_=3.519) | 0.068 | 0.449 | 0.081 |
|  |  | Interaction: GDX*DEX (*F*­_1,40_=1.902) | 0.175 | 0.270 | 0.045 |
|  |  | Interaction: stress*GDX*DEX (*F*­_1,40_=0.122) | 0.729 | 0.063 | 0.003 |
|  |  | Interaction: stress*time (*F*­_1,40_=0.024) | 0.878 | 0.053 | 0.001 |
|  |  | Interaction: DEX*time (*F*­_1,40_=0.123) | 0.728 | 0.063 | 0.003 |
|  |  | Interaction: time*GDX (*F*­_1,40_=0.669) | 0.418 | 0.126 | 0.016 |
|  |  | Interaction: time*stress*DEX (*F*­_1,40_=1.799) | 0.187 | 0.258 | 0.043 |
|  |  | Int.: time*stress*GDX*DEX (*F*­_1,40_=0.454) | 0.504 | 0.101 | 0.011 |
|  |  | Pairwise: SPS GDX time 1 DEX v. veh | <0.0001 | 1.000 |  |
|  |  | Pairwise: SPS GDX time 2 DEX v. veh | 0.015 | 0.697 |  |
|  |  | Pairwise: SPS GDX+T time 1 Dex v. veh | 0.004 | 0.850 |  |
|  |  | Pairwise: SPS GDX+T time 2 Dex v. veh | 0.008 | 0.775 |  |
|  |  | Pairwise: control GDX time 1 DEX v. veh | <0.0001 | 1.000 |  |
|  |  | Pairwise: control GDX+T time 1 Dex v. veh | 0.051 | 0.502 |  |
|  |  | Pairwise: SPS vehicle time 1 GDX v. GDX+1 | <0.0001 | 0.999 |  |
|  |  | Pairwise: control veh time 1 GDX v. GDX+T | <0.0001 | 0.974 |  |
|  |  | Pairwise: SPS DEX GDX time 1 v. time 2 | <0.0001 | 1.000 |  |
|  |  | Pairwise: SPS DEX GDX+T time 1 v. time 2 | 0.009 | 0.764 |  |
|  |  | Pairwise: SPS veh GDX time 1 v. time 2 | <0.0001 | 1.000 |  |
|  |  | Pairwise: SPS veh GDX+T time 1 v. time 2 | <0.0001 | 0.999 |  |
|  |  | Pairwise: control DEX GDX time 1 v. time 2 | <0.0001 | 1.000 |  |
|  |  | Pairwise: control DEX GDX+T time 1 v. time 2 | <0.0001 | 0.997 |  |
|  |  | Pairwise: control veh GDX time 1 v. time 2 | 0.001 | 0.953 |  |
|  |  | Pairwise: control veh GDX+T time 1 v. time 2 | 0.001 | 0.957 |  |
| **PVN (Fig. 4c)** | 2-way ANOVA (stress*GDX)  sham v. GDX | Main effect: stress (*F*­_1,17_=1.532) | 0.233 | 0.215 | 0.083 |
|  |  | Main effect: GDX (*F*­_1,17_=0.560) | 0.464 | 0.109 | 0.032 |
|  |  | Interaction: stress*GDX (*F*­_1,17_=8.396) | 0.010 | 0.780 | 0.331 |
|  |  | Pairwise: SPS GDX v. sham | 0.006 | 0.848 |  |
|  |  | Pairwise: sham SPS v. control | 0.023 | 0.654 |  |
|  | 2-way ANOVA (stress*GDX)  GDX v. GDX+T | Main effect: GDX (*F*­_1,16_=31.424) | <0.0001 | 0.999 | 0.663 |
|  |  | Main effect: stress (*F*­_1,16_=9.759) | 0.007 | 0.834 | 0.379 |
|  |  | Interaction: stress*GDX (*F*­_1,16_=0.183) | 0.675 | 0.069 | 0.011 |
|  |  | Pairwise: SPS GDX v. GDX+T | 0.001 | 0.977 |  |
|  |  | Pairwise: control GDX v. GDX+T | 0.002 | 0.934 |  |
|  |  | Pairwise: GDX SPS v. control | 0.017 | 0.701 |  |
| **Sucrose (Fig. 4e)** | 2-way ANOVA (stress*GDX)  sham v. GDX | Main effect: GDX (*F*­_1,45_=4.200) | 0.046 | 0.518 | 0.085 |
|  |  | Main effect: stress (*F*­_1,45_=3.805) | 0.057 | 0.480 | 0.078 |
|  |  | Interaction: stress*GDX (*F*­_1,45_=1.459) | 0.233 | 0.219 | 0.031 |
|  |  | Pairwise: sham SPS v. control | 0.029 | 0.596 |  |
|  |  | Pairwise: SPS sham v. GDX | 0.027 | 0.608 |  |
|  | 2-way ANOVA (stress*GDX)  GDX v. GDX+T | Main effect: GDX (*F*­_1,38_=2.204) | 0.146 | 0.304 | 0.055 |
|  |  | Main effect: stress (*F*­_1,38_=0.883) | 0.353 | 0.150 | 0.023 |
|  |  | Interaction: stress*GDX (*F*­_1,38_=0.058) | 0.811 | 0.056 | 0.002 |
| **Social int. (Fig. 4d)** | 2-way ANOVA (stress*GDX)  sham v. GDX | Main effect: stress (*F*­_1,52_=6.439) | 0.014 | 0.702 | 0.110 |
|  |  | Main effect: GDX (*F*­_1,52_=0.302) | 0.585 | 0.084 | 0.006 |
|  |  | Interaction: stress*GDX (*F*­_1,52_=0.169) | 0.683 | 0.069 | 0.003 |
|  |  | Pairwise: sham SPS v. control | 0.039 | 0.549 |  |
|  | 2-way ANOVA (stress*GDX)  GDX v. GDX+T | Main effect: stress (*F*­_1,43_=4.314) | 0.044 | 0.528 | 0.091 |
|  |  | Main effect: GDX (*F*­_1,43_=2.710) | 0.107 | 0.363 | 0.059 |
|  |  | Interaction: stress*GDX (*F*­_1,43_=0.683) | 0.413 | 0.128 | 0.016 |
|  |  | Pairwise: GDX SPS v. control | 0.031 | 0.585 |  |
| **Latency/empty zone (Add. file 6g)** | 2-way ANOVA (stress*GDX)  sham v. GDX | Main effect: stress (*F*­_1,52_=0.990) | 0.324 | 0.164 | 0.019 |
|  |  | Main effect: GDX (*F*­_1,52_=0.839) | 0.364 | 0.147 | 0.016 |
|  |  | Interaction: stress*GDX (*F*­_1,52_=0.039) | 0.844 | 0.054 | 0.001 |
|  | 2-way ANOVA (stress*GDX)  GDX v. GDX+T | Main effect: stress (*F*­_1,43_=6.304) | 0.016 | 0.690 | 0.128 |
|  |  | Main effect: GDX (*F*­_1,43_=3.320) | 0.075 | 0.429 | 0.072 |
|  |  | Interaction: stress*GDX (*F*­_1,43_=2.809) | 0.101 | 0.374 | 0.061 |
|  |  | Pairwise: GDX+T SPS v. control | 0.008 | 0.771 |  |
|  |  | Pairwise: SPS GDX v. GDX+T | 0.018 | 0.670 |  |
| **Total social int. (Add. file 6h)** | RM 2-way ANOVA (stress*GDX*target)  Sham vs. GDX | Main effect: target (*F*­_1,52_=97.018) | <0.0001 | 1.000 | 0.651 |
|  |  | Main effect: stress (*F*­_1,52_=1.970) | 0.166 | 0.281 | 0.036 |
|  |  | Main effect: GDX (*F*­_1,52_=0.656) | 0.422 | 0.125 | 0.012 |
|  |  | Interaction: stress*target (*F*­_1,52_=1.329) | 0.254 | 0.205 | 0.025 |
|  |  | Interaction: GDX*target (*F*­_1,52_=0.163) | 0.688 | 0.068 | 0.003 |
|  |  | Interaction: stress*GDX (*F*­_1,52_=0.385) | 0.537 | 0.094 | 0.007 |
|  |  | Interaction: stress*GDX*target (*F*­_1,52_=1.425) | 0.238 | 0.216 | 0.027 |
|  |  | Pairwise: SPS sham target yes vs. no | <0.0001 | .996 |  |
|  |  | Pairwise: Control sham target yes vs. no | <0.0001 | .998 |  |
|  |  | Pairwise: SPS GDX target yes vs. no | <0.0001 | 1.000 |  |
|  |  | Pairwise: Control GDX target yes vs. no | <0.0001 | .973 |  |
|  | RM 2-way ANOVA (stress*GDX*target)  GDX vs. GDX+T | Main effect: target (*F*­_1,43_=81.894) | <0.0001 | 1.000 | 0.656 |
|  |  | Main effect: stress (*F*­_1,43_=0.561) | 0.458 | 0.113 | 0.013 |
|  |  | Main effect: GDX (*F*­_1,43_=0.525) | 0.473 | 0.109 | 0.012 |
|  |  | Interaction: stress*target (*F*­_1,43_=1.231) | 0.273 | 0.192 | 0.028 |
|  |  | Interaction: GDX*target (*F*­_1,43_=0.348) | 0.558 | 0.089 | 0.008 |
|  |  | Interaction: stress*GDX (*F*­_1,43_=1.449) | 0.235 | 0.218 | 0.033 |
|  |  | Interaction: stress*GDX*target (*F*­_1,43_=0.683) | 0.413 | 0.128 | 0.016 |
|  |  | Pairwise: SPS GDX target yes vs. no | <0.0001 | 1.000 |  |
|  |  | Pairwise: Control GDX target yes vs. no | 0.001 | .941 |  |
|  |  | Pairwise: SPS GDX+T target yes vs. no | <0.0001 | .995 |  |
|  |  | Pairwise: Control GDX+T target yes vs. no | <0.0001 | .989 |  |
| **Body weight (Table 3)** | RM 3-way ANOVA (stress*GDX*time) sham v. GDX | Main effect: GDX (*F*­_1,58_=40.087) | <0.0001 | 1.000 | 0.409 |
|  |  | Main effect: time (*F*­_1,58_=340.068) | <0.0001 | 1.000 | 0.854 |
|  |  | Main effect: stress (*F*­_1,58_=1.059) | 0.308 | 0.173 | 0.018 |
|  |  | Interaction: time*stress (*F*­_1,58_=8.048) | 0.006 | 0.797 | 0.122 |
|  |  | Interaction: time*GDX (*F*­_1,58_=39.941) | <0.0001 | 1.000 | 0.408 |
|  |  | Interaction: stress*GDX (*F*­_1,58_=0.240) | 0.626 | 0.077 | 0.004 |
|  |  | Interaction: time*stress*GDX (*F*­_1,58_=1.008) | 0.319 | 0.167 | 0.017 |
|  |  | Pairwise: SPS time 1 sham v. GDX | 0.005 | 0.815 |  |
|  |  | Pairwise: SPS time 2 sham v. GDX | 0.002 | 0.887 |  |
|  |  | Pairwise: SPS sham time 1 v. 2 | <0.0001 | 1.000 |  |
|  |  | Pairwise: SPS GDX time 1 v. 2 | <0.0001 | 1.000 |  |
|  |  | Pairwise: control sham time 1 v. 2 | <0.0001 | 1.000 |  |
|  |  | Pairwise: control GDX time 1 v. 2 | <0.0001 | 1.000 |  |
|  | RM 3-way ANOVA (stress*GDX*time)  GDX v. GDX+T | Main effect: time (*F*­_1,48_=586.955) | <0.0001 | 1.000 | 0.924 |
|  |  | Main effect: stress (*F*­_1,48_=1.696) | 0.199 | 0.248 | 0.034 |
|  |  | Main effect: GDX (*F*­_1,48_=6.459) | 0.014 | 0.702 | 0.119 |
|  |  | Interaction: time*stress (*F*­_1,48_=11.575) | 0.001 | 0.915 | 0.194 |
|  |  | Interaction: time*GDX (*F*­_1,48_=0.491) | 0.487 | 0.106 | 0.010 |
|  |  | Interaction: stress*GDX (*F*­_1,48_=0.016) | 0.900 | 0.052 | 0.000 |
|  |  | Interaction: time*stress*GDX (*F*­_1,48_=0.061) | 0.806 | 0.057 | 0.001 |
|  |  | Pairwise: SPS GDX time 1 v. 2 | <0.0001 | 1.000 |  |
|  |  | Pairwise: SPS GDX+T time 1 v. 2 | <0.0001 | 1.000 |  |
|  |  | Pairwise: control GDX time 1 v. 2 | <0.0001 | 1.000 |  |
|  |  | Pairwise: control GDX+T time 1 v. 2 | <0.0001 | 1.000 |  |
| **Adrenal weight**  **(Table 3)** | RM 2-way ANOVA (stress*GDX)  Sham v. GDX | Main effect: stress (*F*­_1,20_=0.221) | 0.644 | 0.073 | 0.011 |
|  |  | Main effect: GDX (*F*­_1,20_=3.906) | 0.062 | 0.469 | 0.163 |
|  |  | Interaction: stress*GDX (*F*­_1,20_=0.619) | 0.441 | 0.116 | 0.030 |
|  | RM 2-way ANOVA (stress*GDX) | Main effect: stress (*F*­_1,18_=0.399) | 0.535 | 0.092 | 0.022 |
|  |  | Main effect: GDX (*F*­_1,18_=4.357) | 0.051 | 0.506 | 0.195 |
|  |  | Interaction: stress*GDX (*F*­_1,18_=0.885) | 0.359 | 0.145 | 0.047 |
