# Supplementary material for: Sex differences in the traumatic stress response: the role of adult gonadal hormones
Source: Biol Sex Differ. 2018 Jul 13;9:32. doi: 10.1186/s13293-018-0192-8 (PMC6043950; doi:10.1186/s13293-018-0192-8)
Supplement: Supplementary file 6 — Social interaction measures of latency to approach the empty interaction zone and total time spent in social interaction zone. (a) Neither housing nor SPS affected the latency of females to approach the empty rat enclosure, indicating that the effect of SPS is on social interaction per se and not on general activity or exploration of the chamber. (b) Female rats in all groups showed a significant increase in total time spent in the interaction zone when a novel rat was present, regardless of traumatic stress exposure or housing. (c) Sham-operated (intact) control females approached the empty interaction zone more quickly than males, and this effect was independent of SPS. (d) All sham-operated rats spent more time in the interaction zone when a novel rat was present regardless of traumatic stress exposure or sex. (e) SPS had no effect on latency to enter the empty interaction zone, regardless of gonadal status, but gonadectomy (GDX) reduced this latency measure independent of SPS, an effect reversed by T replacement, suggesting that T normally affects overall activity levels in males. (f) All male rats spent more time in the interaction zone when a novel rat was present regardless of traumatic stress exposure or hormone status. (g) Only the combination of SPS and T treatment significantly increased the latency to enter the empty interaction zone in females. (f) All female rats spent more time in the interaction zone when a novel rat was present, regardless of traumatic stress exposure or hormone status. Data presented as mean±SEM. Significance set at P < .05 (*) for planned pairwise comparisons (Bonferroni). See Additional files 2, 3, 4, and 5 for full statistics. (DOCX 109 kb). [file 13293_2018_192_MOESM6_ESM.docx]

**Single-housed vs pair-housed females**

**a b**

**c d**

**e f**

**g h**

24

21

22

23

*

*

*

*

22

23

21

24

**Sham-operated (intact) males vs females**

*

*

*

*

*

15

14

15

14

**Sham-operated vs GDX males**

13

11

*

*

*

*

*

*

*

*

14

11

15

14

**Sham-operated vs GDX females**

*

*

*

*

*

*

*

10

10

14

13

15

14
